# Discovery and Current Status of Evaluation System of Bioavailability and Related Pharmaceutical Technologies for Traditional Chinese Medicines—*Flos Lonicerae Japonicae—Fructus Forsythiae* Herb Couples as an Example

**DOI:** 10.3390/ijms161226132

**Published:** 2015-12-04

**Authors:** Wei Zhou, Baochang Cai, Jinjun Shan, Shouchuan Wang, Liuqing Di

**Affiliations:** 1 College of pharmacy, Nanjing University of Chinese Medicine, Nanjing 210023, China; zhouwei19860506@163.com (W.Z.); bccai@126.com (B.C.); 2 Jiangsu Engineering Research Center for Efficient Delivery System of TCM, Nanjing 210023, China; 3 Nanjing Engineering Research Center for Industrialization of Chinese Medicine Pellets, Nanjing 210023, China; 4 Jiangsu Key Laboratory of Pediatric Respiratory Disease, Institute of Pediatrics, Nanjing University of Chinese Medicine, Nanjing 210023, China; dfsjj@163.com (J.S.); wscnj@126.com (S.W.); 5 Nanjing Haichang Chinese Medicine Group Co., Ltd., Nanjing 210023, China

**Keywords:** bio-pharmaceutics of TCMs preparations, active constitutes identification, evaluation system of bioavailability, pharmaceutical technologies, absorption enhancer

## Abstract

Traditional Chinese medicines (TCMs) have attracted extensive interest throughout the world due to their long history of health protection and disease control, and the internalization of TCM preparations or patented drugs has been considered a wind vane in the process of TCM modernization. However, multi-target effects, caused by multiple components in TCMs, hinder not only the construction of the quality evaluation system (bioavailability), but also the application of pharmaceutical technologies, which results in the poor efficacy in clinical practice. This review describes the methods in the literature as well as in our thoughts about how to identify the marker components, establish the evaluation system of bioavailability, and improve the bioavailability in TCM preparations. We expect that the current study will be positive and informative.

## 1. Introduction

Traditional Chinese medicines (TCMs), utilized in the prevention and treatment of various diseases for thousands of years in China, have been gradually accepted and employed in other countries. TCM preparations or patented drugs, defined by the utilization of herbs, animals, and minerals, with their respective dosages in accordance with the guidance of Chinese medicine theory and the rule of “King, Vassal, Assistant and Delivery servant”, have different dosage forms, such as capsules, tablets, pills, powders, oral liquids, *etc.* [1]. It was reported that the Chinese export of herbal medicines and extracts was significantly higher than that of preparations in the recent years. As shown in 2014, the export of herbal medicines and extracts was worth 2.95 billion dollars, but that of preparations was little, only 250 million dollars [2], which was mainly due to the unsound quality evaluation system (bioavailability) and poor efficacy in clinical practice. For example, Shuang-Huang-Lian oral liquid, a well-known TCM preparation composited of *Flos Lonicerae Japonicae*, *Fructus Forsythiae*, and *Radix Scutellariae*, is usually used as the treatment for acute upper respiratory tract infection caused by bacteria and viruses, but its clinical efficacy was unstable and far lower than that of injection [3]. It was found that multi-target effects, caused by multiple components in TCMs, hindered not only the construction of the evaluation system of bioavailability, but also the formulation, designation, and technologies application. How to establish a quantifiable evaluation system of bioavailability and find suitable pharmaceutical technologies to improve the bioavailability was not only a basic scientific problem of bio-pharmaceutics for TCM preparations, but it was also the key factor in modernizing TCMs.

The following essential problems that refer to the evaluation system of bioavailability construction and pharmaceutical technology applications for TCM preparations exist. Firstly, the network pharmacological effects and the complex structure-effect and dose-effect relationships in TCMs contributed to difficulty in identifying the effective components; Secondly, biological active and pharmacokinetic (absorption, distribution, metabolism, and excretion) diversity of effective constituents resulted in obstacles for setting up weight coefficients for integrating bioavailability; Thirdly, pharmaceutical technologies were hardly applied for TCM preparations due to their complicated physico-chemical properties for both active ingredients and associated constituents.

Therefore, the current problems about how to identify the active components promptly; how to establish a reasonable mathematics model to calculate the weight coefficient to integrate bioavailability; and how to improve the integral bioavailability using related pharmaceutical technologies in TCM preparations need to be further investigated.

## 2. Identification of Active Compounds in Traditional Chinese Medicines (TCMs)

### 2.1. Classic Separation and Analysis

The classic separation and analysis model was performed to identify the active components according to the procedures of extraction, separation, purification, characterization, pharmacological tests, *etc.*, and it was applied to new Chinese herbal monomer or Chinese herbal extract development. For example, artemisinin isolated from the plant *Artemisia annua*, sweet wormwood, and its derivatives possess the most rapid actions against *Plasmodium falciparum* malaria [4]. Digoxin was a purified cardiac glycoside, extracted from *Digitalis lanata*, and was occasionally used to treat various heart diseases, namely atrial fibrillation and atrial flutter [5]. Morphine, a pain medication of the opiate type extracted from *papaver somniferum* L., can decrease feelings of pain through acting directly on the central nervous system (CNS) [6]. Paclitaxel, extracted from the Yew tree, is an anti-cancer drug. It was the first-line treatment for cancers of the breast, colon, lung, *etc.*, and the second-line treatment for AIDS-related Kaposi’s sarcoma [7]. The chemotherapy agent (vincristine), extracted from *Catharanthus roseus*, was utilized as the treatment of leukemias, lymphomas, *etc.* [8]. The total lactones, a Chinese herbal extract from the *Ginkgo* leaf, contained mainly ginkgo lactone A, ginkgo lactone B, ginkgo lactone C, and ginkgo seed lactone, which were prepared as a medicine for preventing or treating deafness and tinnitus [9]. The tea polyphenols, included catechins, theaflavins, tannins, and flavonoids, can prevent coronary heart disease and cancer [10].

### 2.2. Spectrum-Effect Relationships

**Figure 1:**
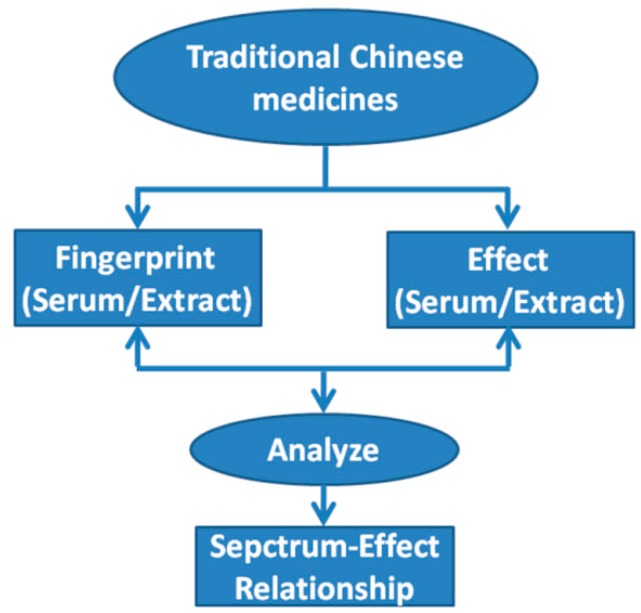
The spectrum-effect relationships for Traditional Chinese medicines (TCMs).

The spectrum-effect relationship, put forward firstly by Li *et al.*, 2002 [11], is an effective method to search for the material foundation of TCMs [12,13,14] via the relationships between TCM fingerprint peaks and specific pharmacodynamic data analyzed by the chemometrics [15], containing hierarchical cluster analysis (HCA), principal component analysis (PCA), the analytic hierarchy process (AHP), stepwise regression analysis (SRA), canonical correlation analysis (CCA), grey relational analysis (GRA), bivariate analysis (BA), multivariate correlation analysis (MCA), *etc.* (Figure 1). As shown in Table 1, there were two spectrum models (*in vitro* chemical fingerprint [16,17,18,19,20,21,22,23,24,25,26,27,28,29,30,31,32,33,34,35,36,37,38,39,40,41,42] and *in vivo* serum fingerprint [43]) analyzed by capillary electrophoresis (CE), infrared spectroscopy (IR) or liquid chromatography (LC) tandem ultraviolet spectrometry (UV), evaporating light scattering detector (ELSD), flow injection chemiluminescence (FICL) and mass spectrometry (MS) [16,17,18,19,20,21,22,23,24,25,26,27,28,29,30,31,32,33,34,35,36,37,38,39,40,41,42,43,44,45], and two pharmacodynamic models (*in vitro* and *in vivo*) in the spectrum-effect relationship. Among them, the chemical fingerprint was obtained from sample preparations for different batches [16,17,18,19,20,21,22,23,24,25,26,27,28,29], different parts [30,31], different combinations [32,33,34], different ways of processing [35,36,37,38,39,40,41], and different agronomic and environmental parameters [42]. For example, Liu *et al.*, 2014 [19], studied the fingerprints of 10 batches of *Radix Astragali* by high performance liquid chromatography (HPLC)-diode array detector (DAD)-ELSD and their anti-gastric ulcer effects evaluated by growth-promoting efficacy in GES-1 cells, and found that ononin, astragaloside III, and astragaloside IV in 16 common peaks were the most correlated with effects by GRA, which provided a theoretical foundation for quality control of *Radix Astragali*. Sun *et al.*, 2013 [30], showed different parts of the fingerprint of *Aconitum* L. plants (*Radix Aconiti Kusnezoffii*, *Radix Aconiti Lateralis Preparata*, and *Radix Aconiti Brachypodi*, *Radix Aconiti*, *Radix Aconiti Singularis*) using ultra-performance liquid chromatography (UPLC)-photodiode array detector (PDA) and demonstrated their anti-bacterial (*Escherichia coli*) activity by micro-calorimetry, and found that hypaconitine and two unknown components (peaks 1 and 3) might be the most important ingredients by using CCA. Bao *et al.*, 2014 [32], reported the fingerprints of 20 combinations in Qizhiweitong granules composed of *Radix bupleuri*, *Rhizoma corydalis*, *Fructus Aurantii*, *Rhizoma cyperi*, *Radix Paeoniae Alba*, and *Radix glycyrrhizae preparata* using HPLC-DAD and their promoting effect on the gastro-intestine evaluated by cyclic guanosine monophosphate (cGMP) and nitric oxide (NO) levels in small intestinal smooth muscle cells, and found that naringin, neohesperidin, hesperidin, neoponcirin, narirutin, liquiritin apioside, albiflorin analogues, neoeriocitrin, and glycyrrhizin were the active components analyzed by the GRA and back propagation (BP) neural network. Zheng *et al.*, 2014 [39], showed the spectrum-effect relationship between the UPLC fingerprint of crude secondary roots of *Aconitumcarmichaelii Debeaux* (FuZi) and its three processed products and their mitochondrial growth (micro-calorimetricmeasurement) analyzed by CCA, and found that benzoylhypacoitine, benzoylaconitine, and mesaconitine might be the main active ingredients. Liu *et al.*, 2014 [43], reported the serum fingerprint at different time points after oral administration of Da-Huang-Fu-Zi-Tang (*Rheum officinale Baill.*, *Aconitum carmichaelii Debx.*, and *Asarum sieboldii Miq.*) by ultra high performance liquid chromatography-electrospray ionization-quadrupole-time of flight-mass spectrometry (UHPLC-ESI-Q-TOF-MS) and their effect on pancreatic acinar cells (AR42J) from injury, and found that rhein isomer methylation, rhein glucoside, hydroxyl-chrysophanol, hypaconine, talatisamine, chysophanol glucuronide conjugation, and chysophanol glucuronide conjugation might be the principle constituents analyzed by CCA.

**Table 1:** Study on the spectrum-effect relationships for Traditional Chinese medicines (TCMs).

Names	TCMs Composition	Fingerprint	Pharmacology	Experimentalmodel	Analytical Method	Active Components	Reference
*Cichorium intybus* L.	-	HPLC-DAD-MS	Anti uric acid	Quails	CCA	Aesculin, chlorogenic acid, chicoric acid, isochlorogenic acid A/B/C and 13,14-seco-stigma5(6), 14(15)-diene-3α-ol	[16]
*Tripterygium glycosides*	*Tripterygium wilfordii*	HPLC	Anti-inflammatory, immunosuppressive activities	mice spleen cells	GRA	Peak 5, peak 10	[17]
*Radix Astragali*	-	HPLC-PDA-ELSD	Anti-gastric effect	Mice, GES-1 cell	GRA	Ononin, astragaloside III, astragaloside IV	[19]
*Rhizoma Coptidis*	-	UPLC-PDA/HPLC-DAD	Antibacterial effect/Anti-MRSA activity/Anti-inflammatory	*Escherichia coli*/Broth microdilution/RAW264.7 mouse macrophage cells	HCA, CCA, PCA, PLS	Berberine, jateorrhizine, palmatine, coptisine, epiberine	[18,20,28]
Da Cheng Qi Tang	*Rhizoma Rhei*, *Cortex Magnoliae officinalis*, *Fructus Aurantii Immaturus*	HPLC-DAD	Purgative effect	Mice	HCA	Hesperidin, aloe-emodin, honokiol, rhein, magnolol, emodin, sennoside A	[21]
*Polygonum cuspidatum*	-	HPLC-DAD-FICL	Anti-oxidant effect	H_2_O_2_ scavenging activities	CA	Piceid, resveratrol, torachrysone-8-*O*-glucoside, questin/physcion, peak1, peak 10	[22]
*Acalypha australis Linn.*	-	UPLC/MS, semi-preparative HPLC	Antibacterial effect	Agar-diffusion method; Broth microdilution method	-	Gallic acid, peak 6, peak 9–11	[23]
*Zathoxylum nitidum*	-	IR	Antitumor effect	7901, Hela cells	MLR	Nitidine chloride	[24]
*Morinanepalensis*	-	HPLC-ELSD	NO inhibition	RAW264.7 cell	PLS	Peak 2, peak 4–6, peak 10, peak 12, peak 13	[25]
*Rheum* species	-	UPLC-PDA	Anti-HIV activity(Ribonuclease H)	enzyme activity	BA	Catechin, epicatechin, aloe-emodinmonoglucoside, Peak (t_R_ = 21.28 min)	[26]
*Rabbiteye blueberry*	-	HPLC-DAD	Antioxidant effect	DPPH radical scavenging	HCA	Delphinins, anthocyanidin-3-glucosides	[27]
EtOAC extracts of *Radix Isatidis*	-	HPLC-DAD	Antibacterial effect	*Escherichia coli*	HCA, MLR, PCA	Salicylic acid	[29]
*Radix Aconiti*, *Radix Aconiti Singularis*, *Radix Aconiti Kusnezoffii*, *Radix Aconiti Lateralis Preparata*, *Radix Aconiti Brachypodi*	-	UPLC-PDA	Antibacterial effect	*Escherichia coli*	CCA	Hypaconitine, peak 1, peak 3	[30]
*Polygonum orientale*	-	UPLC-PDA	Anti-oxidative injury	H9c2 myocardial cell	BA	Peak 3–5, peak 11–14, peak 18, peak 19, peak 21–25	[31]
Qizhiweitong Granules	*Radix bupleuri*, *Rhizoma Corydalis*, *Fructus Aurantii*, *Rhizoma Cyperi*, *Radix Paeoniae Alba*, *Radix glycyrrhizae Preparata*	HPLC-DAD	Promoting gastrointestinal motility	Small intestine smooth muscle cells	GRA, BP neural network	Naringin, neohesperidin, hesperidin, neoponcirin, narirutin, liquiritinapioside, albiflorin analogues, neoeriocitrin, glycyrrhizin	[32]
ZuoJin Wan	*Coptis chinensis Franch.Evodia rutaecarpa (Juss.) Benth.*	HPLC-DAD	Biothermo-logical effect	*Escherichia coli*	CCA	Evodiamine, palmatine hydrochloride, berberine hydrochloride	[33]
Suanzaoren decoction	*Semen Ziziphi Spinosae*, *poria*, *rhizoma Chuanxiong*, *rhizome Anemarrhenae*, *radix glycyrrhizae*	HPLC-PDA	Sedative effect	Mice	Correlation and regressive analysis	Spinosin, ferulic acid, mangiferin, glycyrrhizic acid, peak 3, peak 8, peak 9, peak 16, peak 21, peak 34, peak 42, peak 46, peak 47	[34]
*Platycladi cacumen*	-	HPLC-MS/MS	Hemostatic activities	New Zealand rabbit	CCA	Cecarbon	[35]
*Radix Hedysari*	-	HPLC	Anti-hepatic fibrosis	Mice	GRA, PLS,	Adenosine, calycosin	[36]
*Saffron*	-	HPLC-DAD	Antioxidants	DPPH	MCA	Crocins-1, crocins-2, crocins-3	[37]
*Flos Sophorae*	-	HPLC-MS/MS	Hemostatic activities	New Zealand rabbit	CCA	Huaicarbon A, huaicarbon B	[38]
*Aconitum carmichaelii Debeaus*	-	UPLC-ELSD	Mitochondria growth promoting effect	Rat	CCA	Mesaconitine, benzoylaconitine, benzoylhypacotine	[39]
*Artificial Calculus bovis*	-	UPLC-ELSD	Antibacterial effect	*Escherichia coli*	HCA, MLR, PCA	Cholic acid, taurocholate sodium, chenodeoxycholic acid	[40]
*Belamcanda chinensis* leaf	-	HPLC-DAD	Hypoglycemic effect	Rat	-	Flavonoids (tectoridin, swertisin)	[41]
Da-Huang-Fu-Zi-Tang	*Rheum officinale Baill.*, *Aconitum carmichaelii Debx.*, *Asarum sieboldii Miq.*	UHPLC-ESI-Q-TOF-MS	Anti-acute pancreatitis effect	AR42J cell	CCA	Talatisamine, rhein glucoside, rhein isomer methylation, hypaconine, hydroxyl-chrysophanol, emodin glucuronide conjugation, chysophanol glucuronide conjugation	[43]

CCA: canonical correlation analysis; GRA: grey relational analysis; HCA: hierarchical cluster analysis; PCA: principal component analysis; PLS: partial least squares; MLR: Multiple linear regression ; BA: bivariate analysis ; BP: back propagation; MCA: multivariate correlation analysis; CE: capillary electrophoresis; IR: infrared spectroscopy; LC: liquid chromatography; UV: ultraviolet spectrometry; ELSD: evaporating light scattering detector; FICL: flow injection chemiluminescence; MS: mass spectrometry.

### 2.3. Knock-in and Knock-out

**Figure 2:**
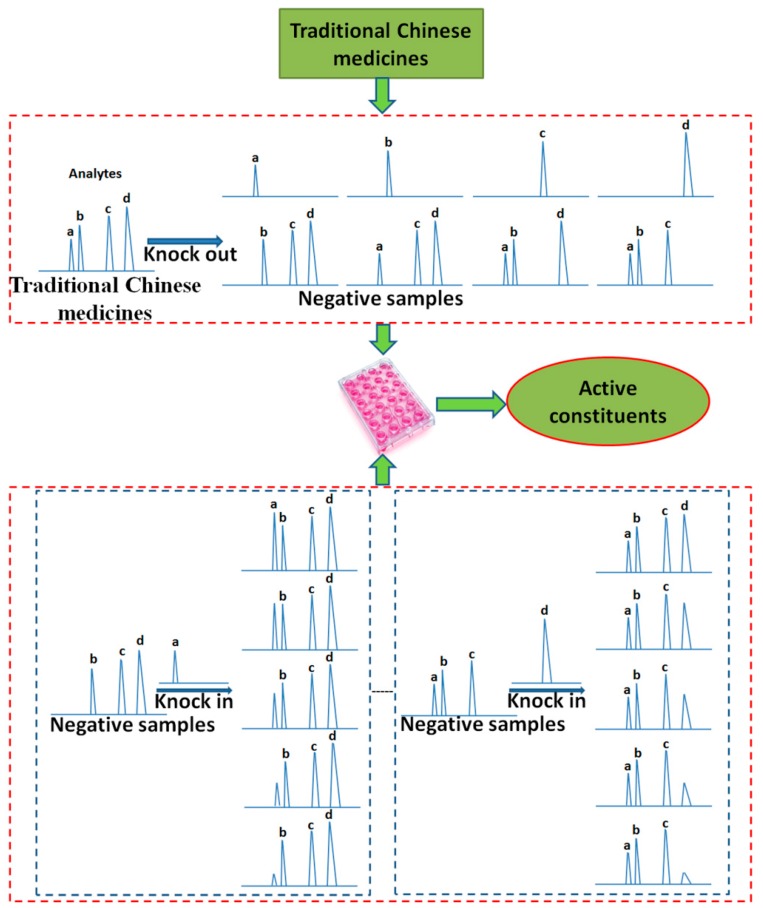
The knock-in/knock-out model for TCMs (a, b, c, and d represent different components in TCMs).

Xiao *et al.*, 2009 [45], put first forward that constituents knock-out/knock-in, inspired by functional genetic methods, are novel patterns of efficient component recognition and quality control for TCMs, which include marker compounds identified by studying the effect of the constituents knocked out on efficacies, and the dosage-effect or dosage-toxicity relationships studied by observing the effect of marker compounds knocked in on efficacies (Figure 2; Table 2). For example, Yan *et al.*, 2014 [46], and Li, 2013 [47], reported the identification of the major active constituents for bacterial diarrhea treatment evaluated by the growth of *Shigella dysenteriae* using microcalorimetry in *Rhizoma coptidis* by the knock-out and knock-in method, and found that coptisine and berberine were the important components with bacteriostatic activities of 54.10% and 39.75%, respectively, by the knock-out method, and their suitable concentration ranged from 8.08% to 31.92% and from 4.05% to 14.45% of the total, respectively, by the knock-in method. Jin *et al.*, 2013 [48], showed the identification of bioactive compounds for osteoporosis treatment evaluated by osteoblasts cell proliferation and differentiation in *Herba Epimedii* by the knock-out method, and found that epimedin A, epimedin B, epimedin C, and icariin were the main active constituents. Yu *et al.*, 2009 [49], studied the assessment of effective components for anti-tumor activity evaluated by the synergistic effects of cyclophosphamide on chemotherapy for S180 tumor-bearing mice in Shenmai formulae composited of *Radix Ginseng* and *Radix Ophiopogonis* by the knock-out method, and found that panoxadiol and a type of ginseoside were the active components.

**Table 2:** Study on the knock-in and knock-out target components in TCMs.

Names	Knock-in or Knock-out Components	Pharmacology	Experimental Model	Active Components	Reference
*Rhizoma Coptidis*	Berberine, palmatine, coptisine, epiberberine, jateorrhizine, columbamine	Growth inhibition of shigelladysenteriae	Microcalorimetry	Berberine, coptisine	[46,47]
*Herba Epimedii*	Epimedin A, epimedin B, epimedin C, icariin	Cell proliferation, differentiation	Third generation rat osteoblasts	Epimedin A, epimedin B, epimedin C, icariin	[48]
*Calculus bovis*	Bilirubin, bilirubin conjugate, glycocholic acid, cholic acid, chenodeoxycholic acid, hyodeoxycholic acid, sodium taurocholic acid, deoxycholic acid	Inhibition of hydrogen peroxide-induced damage	SH-SY5Y	Bilirubin, bilirubin conjugate, glycocholic acid, cholic acid	[50,51]
*Flos Lonicerae Japonicae*	Isochlorogenic acids, chlorogenic acid, flavones, iridoid glycosides	Anti-virus, anti-bacteria	Vero cell, *Escherichia coli*	Isochlorogenic acids	[52]
*Rhizoma Curcumae Longae*	Curcumin, demethoxycurcumin, bisdemethoxycurcumin	Anti-oxidant activity, anti-coagulant effect, anti-oxidant stress damage	DPPH, rabbit, PC12	Curcumin > demethoxycurcumin > bisdemethoxycurcumin	[53,54]
*Radix puerariae*	Puerarin, daidzin, daidzein, compound X	Anti-oxidant damage	HUVEC	Puerarin, compound X	[55]
Shenmai formulae	Panoxadiol, panaxotriol, ophiopogonpolysaccharide, ophiopogonin	Antitumor effect	S180 bearing mice	Panoxadiol, panaxotriol, ophiopogonpolysaccharide	[49]

HUVEC: human umbilical vein endothelial cells; DPPH: 2,2-diphenylpicrylhydrazyl; PC12: pheochromocytoma.

We found above that the knock-in method can be suitable for identifying the effective components in Chinese herbal extracts and Chinese herbal compounds, but the application for the knock-out method is limited due to the fact that the target constituents are difficult to remove from Chinese herbal compounds.

### 2.4. Pharmacokinetics (PK)-Pharmacodynamics (PD)

**Figure 3:**
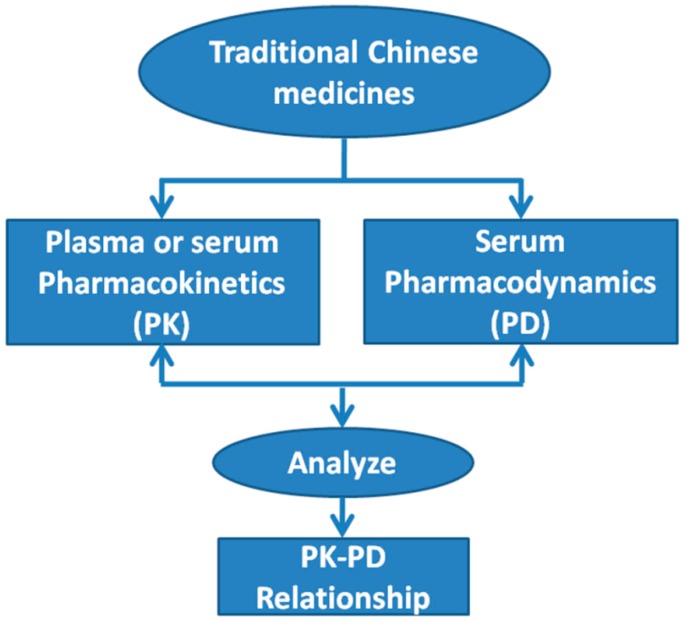
The pharmacokinetics (PK)-pharmacodynamics (PD) relationships for TCMs.

Pharmacokinetics (PK)-pharmacodynamics (PD), put forward first by Sheiner *et al.*, 2009 [56], are extensively applied for effective constituent identification in the field of TCMs, which mainly includes the correlation analysis between PK (the blood-drug concentration method) and PD (the pharmacology-effect method) (Figure 3 and Table 3). For example, Liu *et al.*, 2014 [43], reported the PK profiles of multiple components after oral administration of Da-Huang-Fu-Zi-Tang and the PD profiles evaluated by the effect of the serum at different time points on pancreatic acinar cells (AR42J) from injury, and found that rhein isomer methylation, rhein glucoside, hydroxyl-chrysophanol, hypaconine, talatisamine, chysophanol glucuronide conjugation, and chysophanol glucuronide conjugation might be the principle constituents analyzed by CCA. Peng, 2014 [57], studied the PK of baicalin, geniposide, cholalic acid, hyodeoxycholic acid, chlorogenic acid, and neochlorogenic acid in a Qingkailing injection composed of *Cholalicacid*, *Conchamargaritifera*, *Hyodeoxycholic acid*, *Gardeniae Fructus*, *Cornububali*, *Radix isatidis*, *Baicalin*, and *Flos Lonicerae Japonicae* using UPLC-ESI-MS/MS and studied the PD by evaluating temperature changes in rats, and found that baicalin and geniposide were the main effective ingredients by using Winnonlin software analysis. Wang *et al.*, 2014 [58], showed that Tanshinone IIA was the main ingredient for anti-oxidant activity in a Yin-Teng-Gu-Bi-Kang prescription composed of *Radix Salviae Miltiorrhiae*, *Angelicae Sinensis Radix*, *Paeoniae Radix Alb*, and *Celastrusorbiculatus Thunb.* analyzed by the PK (Tanshinone IIA concentration in plasma)-PD (malondialdehyde (MDA) level in serum) model.

**Table 3:** Study on the pharmacokinetics (PK)-pharmacodynamics (PD) relationships in TCMs.

Names	TCMs Composition	PK Ingredients	PD	Analytical Method	Active Components	Reference
Da-Huang-Fu-Zi-Tang	*Rheum officinale Baill.*, *Aconitum carmichaelii Debx.*, *Asarum sieboldii Miq.*	Talatisamine emodin isomer	Anti-acute pancreatitis effect in AR42J cell	CCA *AUE*-lg*AUC* E-logC Winnonlin	Talatisamin chysophanol glucuronide conjugation	[43]
Qingkailing injection	*Cholalic acid*, *Concha margaritifera*, *Hyodeoxycholic acid*, *Gardeniae Fructus*, *Cornu bubali*, *Radix isatidis*, *Baicalin*, *Flos Lonicerae Japonicae*	Baicalin, geniposide, cholalic acid, hyodeoxycholic acid, chlorogenic acid, neochlorogenic acid	Temperature changes in rat		Baicalin, geniposide	[57]
Yin-Teng-Gu-Bi-Kang Precription	*Radix Salviae Miltiorrhiae*, *Angelicae Sinensis Radix*, *Paeoniae Radix Alb*, *Celastrus orbiculatus Thunb.*	Tanshinone II_A_	MDA in rat’s serum		Tanshinone II_A_	[58]
Shengmai injection	*Red ginseng*, *ophiopogon japonicas (Thunb.) Ker-Gawl*, *schisandra chinensis*	Ginsenoside (Rg1, Rb1)	NO in rat’s serum		Ginsenoside (Rg1, Rb1)	[59]
*Rhizoma Curculiginis*	—	Orcinol glucoside	SOD, GSH, GSH-PX in plasma		Orcinol glucoside	[60]
*Schisandra chinensis* alcoholic extract	—	Schisandrin, gomisin D, gomisin O, tigloylgomisin H, angeloylgomisin Q, gomisin G, gomisin B, angeloylgomisin P, schisantherin A, gomisin E, schisantherin D, deoxyschizandrin, gomisin R, γ-schisandrin, angeloylisogomisin O, angeloylgomisin O, 6-*O*-benzoyl gomisin O, 7-8-dihydroxy-schizandrin, Peak_tR_ (42.0 min)	ALT in rat’s serum		Schisandrin, schisantherin A, deoxyschizandrin, γ-schisandrin, 7-8-dihydroxy-schizandrin, Peak_tR_ (42.0 min)	[61]
*Radix et Rhizoma Rhei*	—	Aloe Emodin, rhein, emodin, chrysophanol	Amylase, endotoxin, TNF-α, diamineoxidase in beagle dog’s serum; Temperature changes and NO in rat *in vivo*		Rhein	[62]
Tea polyphenols	—	Epigallocatechingallate, epicatechingallate, epigallocatechin, epicatechin	MDA in rat’s liver		Epigallocatechi-n gallate, epicatechingallate, epigallocatechi-n, epicatechin	[63]

SOD: superoxide dismutase; GSH: glutathione; GSH-PX: glutathione peroxidase; ALT: alanine transaminase; MDA: malondialdehyde; *AUE*: area under efficacy; *AUC*: area under concentration. E: efficacy; C: concentration.

## 3. Evaluation System of Bioavailability Establishment for TCMs

**Figure 4:**
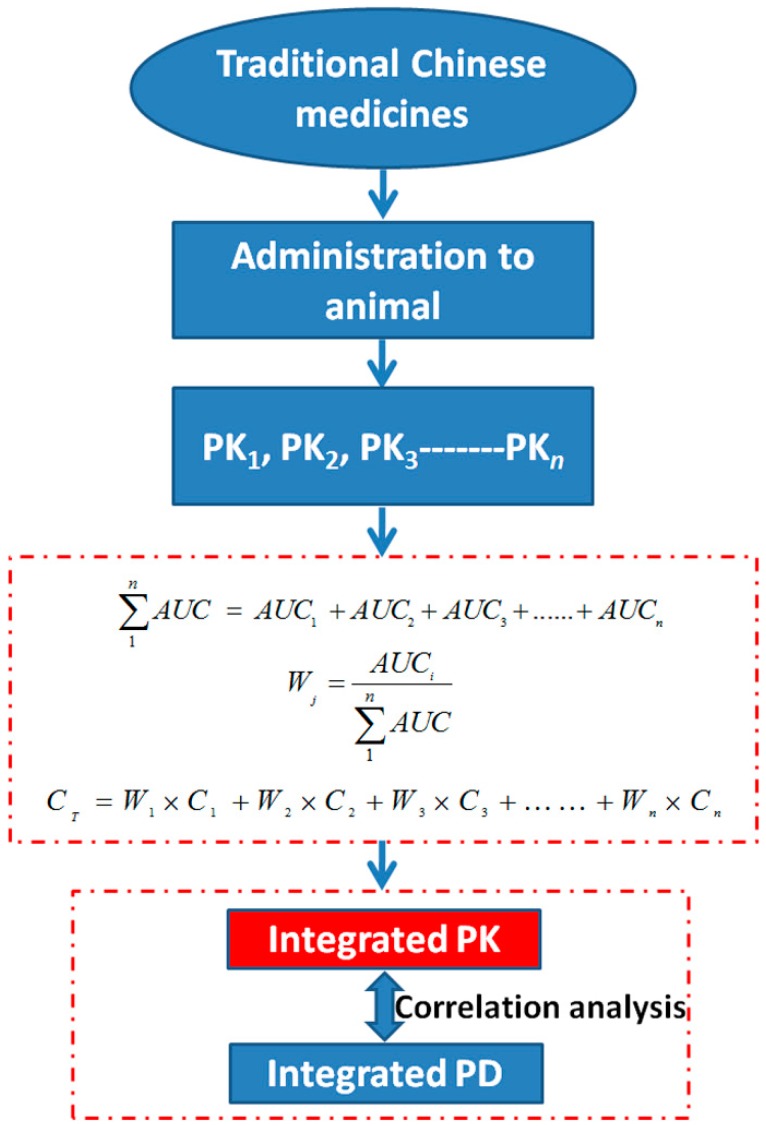
The integrated pharmacokinetics for TCMs.

The construction of the evaluation system of bioavailability is one of the most important scientific issues in the modernization of TCMs. Hao *et al.*, 2009 [64], first reported that an area under curve (*AUC*)-weighting method could obtain the integral PK properties based on the same type of components in TCMs (Figure 4). The weighting coefficient for each constituent was calculated using Equations (1) and (2). The integral concentrations (*C_T_*) at each time point were then calculated by Equation (3), where *w* represented the weighting coefficient, *AUC*_1_–*AUC_n_* represented bioavailability *in vivo* and *C*_1_–*C_n_* represented the plasma concentration of each constituent studied. The evaluation system establishment could comprehensively estimate the correlation between integral PK and PD, especially for the TCMs with a narrow therapeutic window, to ensure safety in practical applications. As seen in Table 4, Dong *et al.*, 2014 [65], showed the integral PK profiles of Rhodojaponin I, II, and III, the active components in *Rhododendri Mollis Flos*, and found that the correlation with the potential markers of myocardial injury ((creatine kinase-measurement blood) (CK-MB) and lactate dehydrogenase (LDH)) was fairly strong, which can be conductive to fully understanding the relationship between the PK behaviors and the compound’s efficacy. Guo *et al.*, 2014 [66], and Li *et al.*, 2008 [67], successfully developed the integral PK profiles in the plasma and brain of ginsenosides Rg1, Rb1, Re, Rd, and panax notoginsenoside R1, the main active components in *Panax notoginseng* (Burk.) *F.H.Chen* (Sanqi). Pan *et al.*, 2014 [68], and Zhu *et al.*, 2012 [69], showed the integrated PK of baicalin, baicalein, geniposide, palmatine, and berberine, the main effective ingredients in Huang-Lian-Jie-Du-Tang in middle cerebral artery occlusion (MCAO) rats, and found that the correlation with the anti-ischemia index (Interleukin 6 (IL-6), tumor necrosis factor (TNF-α), superoxide dismutase (SOD), glutamic acid (Glu), and MDA) in the serum was good, which would provide comprehension better understanding of cerebrovascular disease as Huang-Lian-Jie-Du-Tang is used in clinical practice. Xie *et al.*, 2010 [70], reported the holistic PK of Schisandrin, schisantherin A, deoxyschisandrin, and γ-schisandrin, the four main lignin components in *Schisandra*, and found that the integral *AUC* and CYP3A activities correlated well with hepatic injury biomarkers (ALT and aspartate aminotransferase (AST)) in serum. However, the integral PK calculated by an *AUC*-weighting method was established on the basis of the fact that the bioavailability of the integral components was positively correlated with their efficacy. For example, the *AUC* value of compound A was higher than that of compound B, but their efficacy was opposite. It means that the effect of the bioavailability fluctuation of compound B on the integral *AUC* was far less than that of compound A, but that its effect on the pharmacology was far stronger than that of compound A, which resulted in the integral PK parameters being negatively or not correlated with pharmacology. Therefore, an *AUC*-weighting method might not be well suited for studying the integral PK of all TCMs. It was presumed that efficacy as a weight coefficient might be more reasonable if the efficacy we chose could represent the pharmacological effects of TCMs.

(1) $$\sum_{1}^{n}AUC\;=\;AUC_{\text{1}}\;+AUC_{2}+AUC_{\text{3}}+......+AUC_{n}$$

(2) $$W_{j}=\frac{AUC_{i}}{\sum_{1}^{n}AUC}$$

(3) $$C_{T}=W_{1}\times C_{\text{1 }}+W_{2}\times C_{\text{2}}+W_{3}\times C_{\text{3}}+\ldots \ldots +W_{n}\times C_{n}$$

**Table 4:** Study on the integrated pharmacokinetics in TCMs.

Names	TCMs Composition	Integrated Ingredients	Integrated Method	Pharmacology	Correlation Analysis	Reference
*Rhododendri Mollis Flos*	-	Rhodojaponin (I, II, III)	Weighting factor based on *AUC*	Myocardial damage (LDH, CK-MB)	-	[65]
*Panax Notoginseng* Saponins1	-	Panax Notoginsenoside R1, Ginsenosides Rg1, Rb1, Re, Rd		-	-	[66,67]
Huanglian-Zhizi couplet medicine	*Rhizoma Coptidis*, *Fructus Gardeniae*	Gardenia acid, geniposide		Antioxidant efficacy (SOD)	*E*-*C*	[68]
Huang-Lian-Jie-Du-Tang	*Rhizomacoptidis*, *Radix scutellariae*, *Cortex phellodendri*, *Fructusgardeniae*	Berberine, palmatine, baicalin, baicalein, geniposide		Anti-ischemia	-	[69]
*Schisandra* lignans	-	Schisandrin, schisantherin A, deoxyschisandrin, γ-schisandrin		Serum alanine aminotransferase (ALT), aspartate aminotransferase (AST)	*E*-*C*	[70]
Jiao-Tai-Wan	*Rhizomacoptidis powder*, *Cortex cinnamomi* powder	Berberine, palmatine, coptisine, epiberberine, jatrorrhizine		-	-	[71]
Huang-Lian-Jie-Du-Tang	*Rhizomacoptidis*, *Radix scutellariae*, *Cortex phellodendri*, *Fructusgardeniae*	Groenlandicine, berberine, palmatine, epiberberine, jatrorrhizine, columbamine		-	-	[72]
Total coumarins in *Radix Angelicae dahuricae*	-	Bergapten, imperatorin…isoimperatorin		-	-	[73]
Tea polyphenols	-	Epigallcocatechingallate, Epicatechingallate, Epigallocatechin, Epicatechin		Anti-lipid peroxidation *in vitro* of mouse liver homogenate	*E*-log*C*	[74]
Gegen-Qinlian Decoction	*Radix Puerariae*, *Radixscutellariae*, *Coptidisrhizome*, *Radixglycyrrhizae*	Puerarin, Daidzein, Baicalin, Baicalein, Wogonoside, Wogonin, Glycyrrhizin, Liquiritin, Berberine, Jateorhizine, Palmatine		-	-	[75]

LDH: lactate dehydrogenase; CK-MB: creatine kinase-measurement blood; *E-C*: effect-concentration.

## 4. Pharmaceutical Technology Applications to Improve the Bioavailability of Active Components in TCMs

**Figure 5:**
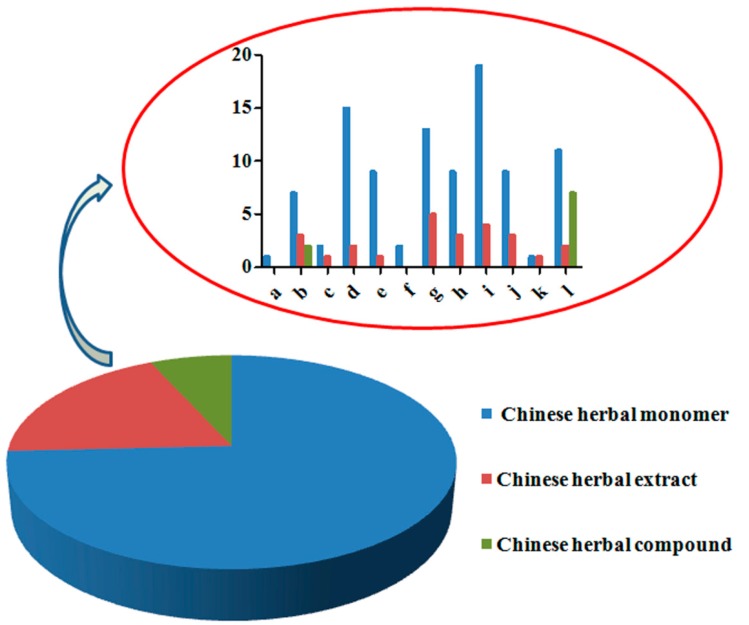
The applications of related pharmaceutical technologies for Chinese herbal monomers, extracts and compound. (a: bioadhesive polymers; b: mircoemulsion; c: self-microemulsion; d: nanosuspensions; e: polymer β-Cyclodextrin inclusion; f: polymeric micelles; g: liposomes; h: phospholipid complex; i: solid lipid nanoparticles; j: solid dispersion; k: micronization; l: absorption enhancer).

As we all know, the low oral bioavailability of TCMs will contribute to their poor clinical therapeutic effects. However, formulation designation and pharmaceutical technology applications are severely disrupted by the complex physico-chemical properties for both active ingredients and their associated constituents in TCMs. As reported in the PubMed Database (2006–current), the pharmaceutical methods applied to TCMs classified II (high permeability and low solubility) in the Biopharmaceutics Classification System (BCS) [76] included mainly micronization [77,78], nano-suspensions [79,80,81,82,83,84,85,86,87,88,89,90], solid dispersion [91,92,93,94,95,96,97,98,99,100,101,102,103,104,105,106,107,108], phospholipid complex [92,109,110,111,112,113,114,115,116,117,118,119,120], β-cyclodextrin complex [121,122,123,124,125,126,127,128,129,130,131], microemulsion [132,133,134,135,136,137,138,139,140,141,142,143,144,145], self-microemulsion [146,147,148], and polymeric micelles [149,150], *etc.* The pharmaceutical methods applied to TCMs classified III (low permeability and high solubility) [76] included mainly microemulsion [132,133,134,135,136,137,138,139,140,141,142,143,144,145], liposome [151,152,153,154,155,156,157,158,159,160,161,162,163,164,165,166,167,168,169,170,171,172], lipid nanoparticles [173,174,175,176,177,178,179,180,181,182,183,184,185,186,187,188,189,190], bioadhesive polymer [191], absorption enhancers [192,193,194,195,196,197,198,199,200,201,202,203,204,205,206], *etc.* According to the statistics, the proportions of the pharmaceutical technologies applied to Chinese herbal monomers, Chinese herbal extracts, and Chinese herbal compounds, respectively, were 74.24%, 18.94%, and 6.82% (Figure 5); the percentage of Chinese herbal compounds using absorption enhancers was 77.78% compared with those using other methods (Figure 5), which indicated that the absorption enhancers should be considered the preferred pharmaceutical technology in Chinese herbal compound preparations, such as the preparations recorded in *Chinese Pharmacopeia* (Volume I) [207] as the active constituents recognized as belonging to those classified III in the BCS [76].

## 5. Study on the Evaluation System of Bioavailability Establishment and Related Pharmaceutical Technologies—*Flos Lonicerae Japonicae—Fructus Forsythiae* Herb Couples as an Example

**Figure 6:**
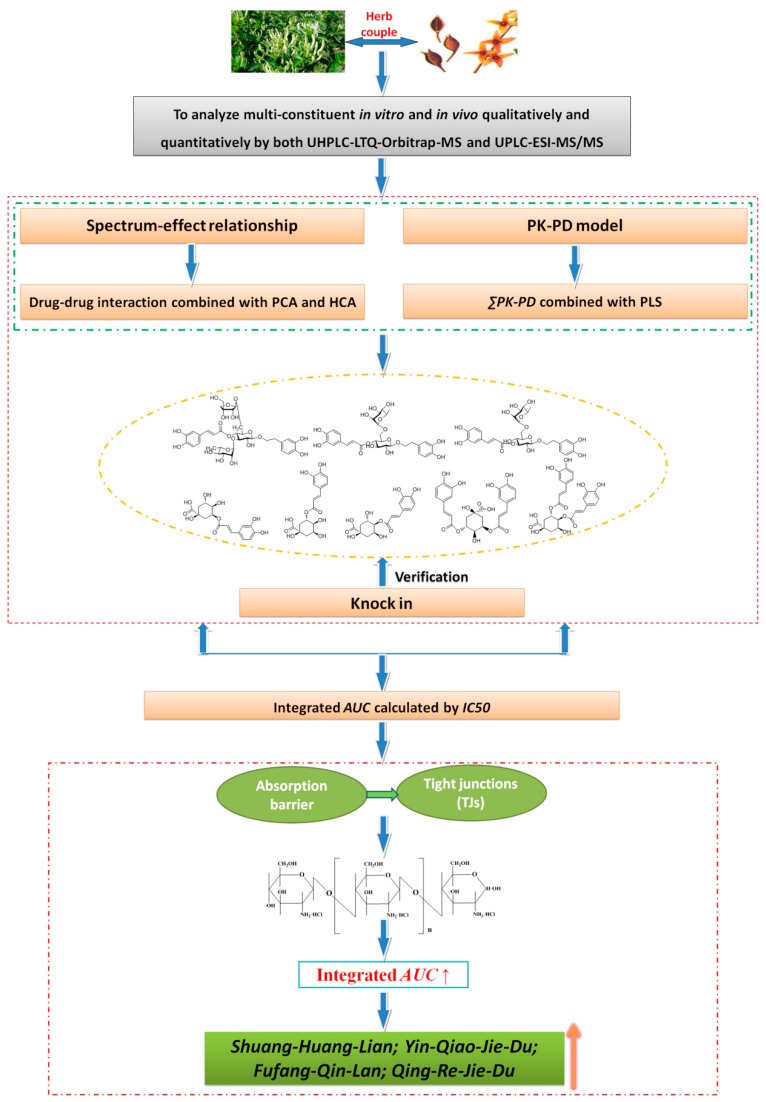
The evaluation system of bioavailability establishment and related pharmaceutical technology applications for the *Flos Lonicerae Japonicae*-*Fructus Forsythiae* (FLJ-FF) herb couple (↑: improvement).

We have previously studied the evaluation system of bioavailability establishment and related pharmaceutical technology applications based on the *Flos Lonicerae Japonicae*-*Fructus Forsythiae* (FLJ-FF) herb couple as a model drug (Figure 6).

Firstly, the qualitative and quantitative methods *in vitro* and *in vivo* for the multi-constituents in the FLJ-FF herb couple were established. We found 35 components *in vitro* using UHPLC-LTQ-Orbitrap-MS, including seven phenolic acids, five phenylethanoid glycosides, seven flavones, two isoflavones, nine lignans, two saponins, and three iridoids, and 26 ingredients (neochlorogenic acid, chlorogenic acid, cryptochlorogenic acid, 3,5-dicaffeoylquinic acid, 3,4-dicaffeoylquinic acid, caffeic acid, quinic acid, isoforsythoside, forsythoside A, forsythoside B, rutin, luteolin, astragalin, hyperoside, isoquercitrin, quercetin, luteoloside, genistin, genistein, arctiin, phillyrin, pinoresinol-β-d-glucoside, arctigenin, dipsacoside B, macranthoidin B, and loganin) were quantified simultaneously by UPLC-ESI-MS/MS [208]. Meanwhile,32 components *in vivo* (29 prototype compounds and three metabolites) were identified by UHPLC-LTQ-Orbitrap-MS with MetWorks software, which included seven phenolic acids, five phenylethanoid glycosides, seven flavones, two isoflavones, seven lignans, one iridoid, and three metabolites (pinoresinol-*O*-glucuronide, epipinoresinol-*O*-glucuronide, and phillygenin-*O*-glucuronide), and 23 ingredients (neochlorogenic acid, chlorogenic acid, cryptochlorogenic acid, 3,5-dicaffeoylquinic acid, 3,4-dicaffeoylquinic acid, caffeic acid, quinic acid, isoforsythoside, forsythoside A, forsythoside B,rutin, luteolin, astragalin, hyperoside, isoquercitrin, quercetin, luteoloside, genistin, genistein, phillyrin, pinoresinol-β-d-glucoside, and arctigenin) were quantified simultaneously by UPLC-ESI-MS/MS [209].

Secondly, both drug-drug interaction (DDI) (spectrum-effect relationship) [208] and ΣPK-PD (PK-PD model) [210] were simultaneously performed to identify the chemical markers in the FLJ-FF herb couple, and they were verified by the “knock-in” method (data not shown). The result showed that caffeic acid derivatives(neochlorogenic acid, chlorogenic acid, cryptochlorogenic acid, 3,5-dicaffeoylquinic acid, 3,4-dicaffeoylquinic acid, isoforsythoside, forsythoside A, forsythoside B) can be considered as marker compounds in the FLJ-FF herb couple.

Thirdly, the integral PK for caffeic acid derivatives based on an *AUC*-weighting approach was established, but the bioavailability of chlorogenic acids was negatively correlated with their efficacy. For example, the *AUC* of chlorogenic acid in the FLJ-FF herb couple was much higher than that of forsythoside A, but the *IC*50 was lower than that of forsythoside A (data not shown). It meant that the effect of the bioavailability fluctuation of phenylethanoid glycosides on the integral *AUC* calculated by an *AUC*-weighting approach was far less than that of the chlorogenic acids, but the effect on the pharmacology was far more than that of the chlorogenic acids, which resulted in the antiviral activity, not the integral *AUC*, being improved significantly as forsythoside A knocked in the FLJ-FF herb couple (data not shown). However, the integral *AUC* calculated by *IC*50 as follows (W represents the weighting coefficient and the *C*_1_–*C_n_* represents the plasma concentration of the components studied) was increased gradually as the antiviral activity was improved by the FLJ-FF herb couple knocked-in forsythoside A, showing a strong positive correlation, and the integral PK parameters using *IC*50 as the weight coefficient index could fully take eight caffeic acid derivatives’ PK parameters into account (data not shown). The results above indicated that *IC*50 as a weight coefficient was more reasonable than *AUC.*

(4) $$\sum_{1}^{n}\frac{\text{1}}{IC50}=\frac{1}{IC50_{1}}+\frac{1}{IC50_{2}}+\frac{1}{IC50_{3}}+\ldots \ldots +\frac{1}{IC50_{n}}$$

(5) $$W_{j}=\frac{\frac{\text{1}}{IC50_{j}}}{\sum_{1}^{n}\frac{1}{IC50}}$$

(6) $$C_{T}=W_{1}\times C_{\text{1 }}+W_{2}\times C_{\text{2}}+W_{3}\times C_{\text{3}}+\ldots \ldots +W_{n}\times C_{n}$$

Finally, the antiviral activity of commercially available FLJ-FF herb couple preparations (Shuang-Huang-Lian oral liquid, Yin-Qiao-Jie-Du tablet, Fufang-Qin-Lan oral liquid, and Qing-Re-Jie-Du oral liquid) was regulated based on the integrated *AUC* calculated by *IC*50. The antiviral effect was decreased significantly as the four preparations knocked out the FLJ-FF herb couple, but increased significantly as the FLJ-FF herb couple was knocked in (data not shown). Besides, the integral absorption of caffeic acid derivatives in the four preparations was improved significantly both *in vitro* and *in vivo* by the chito-oligosaccharide (COS) (data not shown), which was consistent with the fact that the absorption of caffeic acid derivatives in monomers, the FLJ-FF herb couple, or its preparations was mainly restricted by tight junctions (TJs) [211,212,213,214], and COS was an absorption enhancer based on tight junctions with high effectiveness and low mucosal toxicity [215]. In addition, the treatment with FLJ-FF herb couple preparations with COS can restrain the MDCK cell damage upon influenza virus propagation better than that of the control [216], but the treatment with the preparations with the COS-knocked-out FLJ-FF herb couple showed non-significance compared to that of control (data not shown). The results above illustrated not only the antiviral activity improvement due to the COS in FLJ-FF herb couple preparations resulting from the improvement of the integrated AUC of caffeic acid derivatives, but also showed the reasonability of the weight coefficient calculated by *IC*50, not AUC. Absorption-enhancer COS has been successfully applied for the second development of FLJ-FF herb couple preparations.

## 6. Conclusions and Future Perspective

**Figure 7:**
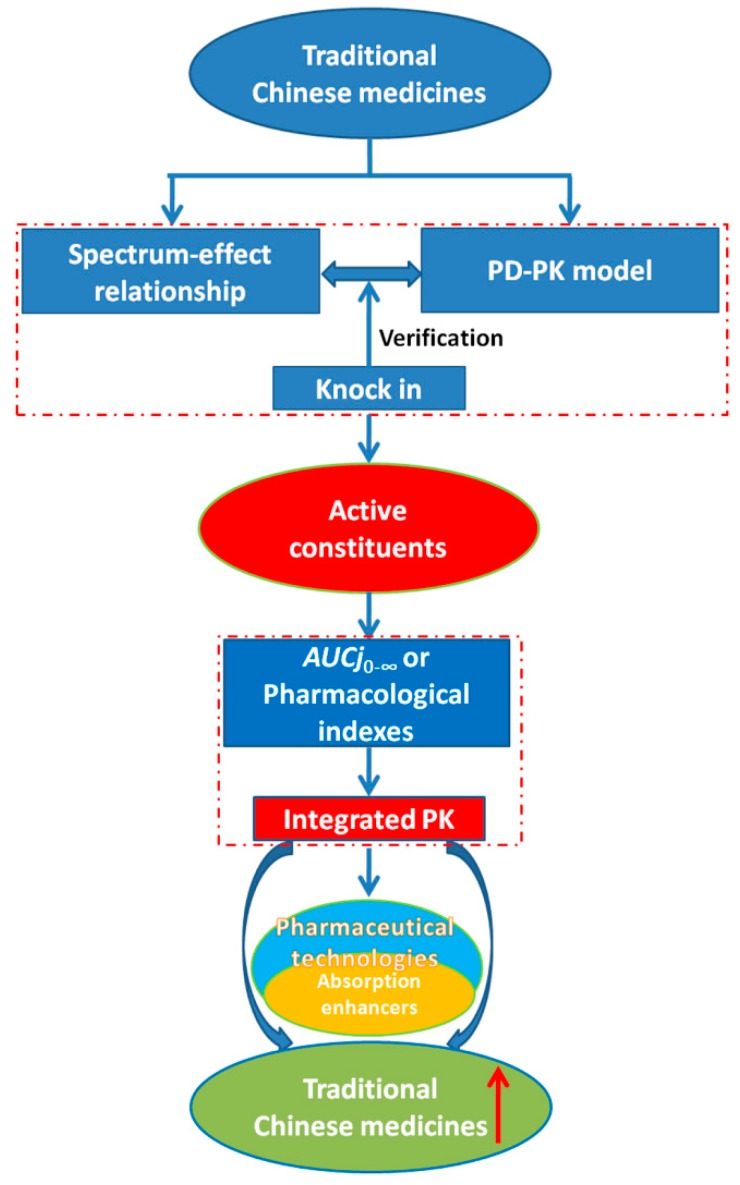
The path for studying bio-pharmaceutics for TCM preparations (↑: improvement).

TCM preparations, extensively recorded in Chinese Pharmacopoeia, have long history with applications for protecting health and controlling disease [207]. The present quality assessment of TCM preparations mainly focused on single chemical constituents, not biological indicators, as markers, and novel pharmaceutical excipients were hardly applied for TCM preparations due to their complicated physico-chemical properties, which resulted in poor effects in clinical practice. Here, we attempted to propose a plan (Figure 7) to deal with the obstacles in order to carry out the bio-pharmaceutical explorations of TCM preparations better. Firstly, both the spectrum-effect relationship and PK-PD model can be simultaneously performed to identify the chemical markers and to be verified by the “knock-in” method; Secondly, the weight coefficient calculated by *AUC* or the efficacy should be compared to decide which one is more suitable for the integral PK; Thirdly, an absorption enhancer might be considered the preferred pharmaceutical technology in Chinese herbal compound preparations, such as the preparations recorded in Chinese Pharmacopeia (Volume I) as the active constituents recognized as belonging to those classified III in the BCS [207].

In recent years, LC-MS was rapidly accepted by the analytical community, and it was gradually applied for qualitative and quantitative analysis [217,218,219,220], PK study [221,222], metabolite *in vivo* identification [223,224], metabolomics [225,226], quality control [227,228], and pharmacological studies [229,230] in TCMs. The novel methods, such as aggregation morphology [231] and magnetic molecularly imprinted polymer [232], were also helpful in understanding the mechanism of TCMs and discovering drugs based on TCMs. Besides, systems biology (genomics, proteomics, metabolomics, and bioinformatics), a new subject in the field of life sciences, provided a comprehensive resource for the modernization and advancement of TCMs as well as general drug discovery efforts, which proposed a system-to-system research methodology to study the interaction between TCMs and the human body and their applications in drug research and development [233]. In addition, some promising excipients can also accelerate the development of TCM preparations. For example, Kollidon CL, manufactured by BASF, the largest chemical producer in the world, can produce the highest disintegration speed (18 min), which is 50% faster than croscarmellose sodium (CMC-Na: 27 min) and almost three times faster than sodium starch glycolate (CMS-Na: 50 min), and which can be applied for surmounting the obstacles of poor solubility and long disintegration times in oral solid dosage forms of TCM preparations such as tablets or capsules when the active constituents recognized belonged to those classified II in the BCS. Chitosan derivatives, such as *N*-trimethyl chitosan chloride [234] and chito-oligosaccharide [215], synthesized with remarkable solubility at neutral pH in an aqueous environment, were not only non-toxic, biocompatible, and biodegradable, but also performed as intestinal absorption enhancers by reversible opening of the tight junctions, which can be applied for improving the permeability of active constituents as the active constituents recognized belonged to those classified III in the BCS. We expect that the current study will be positive and informative.
